# Enhancing grid reliability through advanced insulator defect identification

**DOI:** 10.1371/journal.pone.0307684

**Published:** 2024-09-26

**Authors:** Xiao Wang, Ting Yang, Yuntao Zou

**Affiliations:** 1 School of Television Arts, Communication University of Zhejiang, Hangzhou, China; 2 Hubei Huazhong Electric Power Technology Development Co., Ltd, Wuhan, China; 3 School of Energy and Power Engineering, Huazhong University of Science and Technology, Wuhan, China; Sichuan University, CHINA

## Abstract

The article presents an innovative approach for detecting defects in insulators used in high-voltage power transmission lines, employing an enhanced Detection Transformer (DETR) model, termed IF-DETR. The study addresses the significant challenges in traditional insulator defect detection methods, such as the loss of small defect features and confusion with background features. Firstly, we propose a multi-scale backbone network to better extract features of small objects. Secondly, as the contextual information surrounding objects plays a critical role in detecting small objects, we introduce a fusion module composed of ECA-Net and SAU to replace the original attention module for improved contextual information extraction. Lastly, we introduce the insulator defect (IDIoU) loss to optimize the instability in the matching process caused by small defects. Extensive experiments demonstrate the model’s effectiveness, particularly in detecting small defects, marking a notable advancement in insulator defect detection technology. The IF-DETR achieved a 2.3% increase in AP compared to existing advanced methods. This method not only enhances the accuracy of defect detection, crucial for maintaining the reliability and safety of power transmission systems but also has broader implications for the maintenance and inspection of high-voltage power infrastructure.

## 1. Introduction

Insulators in high-voltage power transmission are components used to support and secure transmission lines. Their primary function is to support conductors on transmission lines while preventing the current from flowing through the supporting structures into the ground or other objects that should not be electrified. Insulators are typically made from materials such as ceramics, fiberglass-reinforced plastics, or rubber to ensure they can effectively prevent current leakage. Additionally, insulators can prevent the formation of electrical arcs caused by high voltages, which could damage transmission equipment and lead to power transmission interruptions. In high-voltage power transmission, insulators play a crucial role in ensuring the safe operation of power lines.

Damage to insulators can lead to a series of severe consequences. First, there is the interruption of power transmission; damaged insulators can cause a short circuit in power lines, leading to transmission interruptions. Second, there are safety risks; if an insulator is damaged, the current may flow down the pole to the ground, increasing the risk of electrocution. Furthermore, damaged insulators can cause electrical arcs, posing a fire hazard. Third, there are economic losses; failures in transmission lines can disrupt the power supply to businesses and households, leading to corresponding economic losses. Finally, short circuits caused by damaged insulators can damage electrical equipment. Even if there is no immediate apparent equipment failure, it can lead to wear and tear on power system equipment over time, reducing equipment life and posing risks to the stability of the power system. Therefore, maintenance and inspection of insulators are important measures to ensure the safe and stable operation of high-voltage power transmission systems.

Insulators are key electrical components in overhead transmission systems, bearing the critical responsibility of preventing current leakage and maintaining line stability. Unfortunately, these devices may suffer from self-explosion, breakage, or flashover issues due to pollution, as they are constantly exposed to strong electric fields and harsh environmental conditions. Specifically, surface failures caused by contamination and their damage to small insulators have become a leading cause of power grid failures. It is estimated that such defects account for over half of all grid failures. Therefore, developing methods for the rapid and accurate detection of insulator conditions is essential for ensuring the stable operation of power grids.

With the development of drone and computer vision technology, optical remote sensing images, including drone imagery, have become increasingly important in both military and civilian applications [1]. The ability to locate and identify objects in these images is a fundamental challenge in image processing, with wide-ranging implications for disaster monitoring, urban management, and precision control [2–4]. Currently, traditional detection methods that rely on helicopter or manual field inspections, as well as the analysis of drone-captured images, are inefficient and costly in the face of China’s complex power grid structure. This is particularly true for the identification of small defect targets in drone images, where the complex background increases the difficulty of recognition. Given this, the development of a technology suitable for detecting small target faults in complex backgrounds is urgently needed to enhance the efficiency and accuracy of detection efforts.

In recent years, DEtection TRansformer (DETR) [5] has made significant progress in the field of object detection, which has revolutionized object detection by treating it as a set prediction task and leveraging the powerful relational modeling capabilities of the Transformer. Unlike traditional methods, DETR directly assigns object queries to real targets without the need for post-processing steps. However, DETR still faces challenges such as slow convergence and lower performance in detecting small objects. While researchers have made efforts to address the slow convergence, the challenge of detecting small objects persists.

Although DETR variants offer numerous advantages, they also face several challenges when applied to the detection of insulator defects. Firstly, as deep networks progress, the scale of feature layers diminishes, resulting in the gradual loss of small defect features. This loss of information limits the model’s ability to accurately detect minor defects. Secondly, the self-attention operations in DETR tend to overpower the limited information present in small defects, causing contamination from background features. This issue leads to decreased detection accuracy. Lastly, the Hungarian matching algorithm, which is commonly used in DETR, has inherent shortcomings. Minor shifts in the predicted bounding boxes can cause significant fluctuations in Intersection over Union (IoU) [6], making the optimization process difficult.

To addresses these challenges, we proposed the IF-DETR. There are two innovations in this model, one is the fusion of low-level features from the backbone network with high-level features from the Transformer through the fusion module of ECA-Net and SAU to further capture contextual information. The other is to solve the instability problem caused by small defects in the matching process through IDIoU loss function and to speed up the training.

By incorporating these improvements into DETR variants, we aim to enhance the detection performance of insulator defects, particularly minor defects. The experimental results demonstrate the effectiveness of our proposed methods and highlight the potential for their application in real-world scenarios. Ultimately, these advancements contribute to the evolving field of object detection and pave the way for more accurate and efficient detection of insulator defects.

The remainder of this paper is structured as follows. Section 2 describes the related work in traditional object detection and Transformer-based object detection methods. Section 3 describes the overall structure of the Insulator Defect DETR model and the details of the proposed methods. Section 4 presents the experimental results. Section 5 discusses the ablation and generalization studies of the IF-DETR model. Section 6 concludes the contents of this work.

## 2. Related work

### 2.1 CNN-based methods

Deep learning methods, particularly Convolutional Neural Networks (CNN), have shown great promise in object detection models for remote sensing images [7, 8]. These models can be broadly classified into two categories: anchor-based approaches and anchor-free approaches.

Anchor-based approaches involve manually setting anchor sizes, which can lead to redundancy in computation and memory load [9]. While these methods have shown good performance, the reliance on manually defined anchors can be limiting in certain scenarios. On the other hand, anchor-free approaches directly provide object location and class information, eliminating the need for anchor adjustment [10]. By converting the traditional bounding box prediction problem into a corner prediction problem, anchor-free approaches avoid unnecessary hyperparameters and streamline the detection process.

In addition to these approaches, transformer-based detection methods have emerged as a dynamic and focused alternative. These methods do not require predetermined anchors or post-processing techniques like Non-Maximum Suppression (NMS) [11]. Instead, transformer-based models directly output prediction results, making them more efficient and accurate [12].

The YOLO series models have been widely used in CNN-based object detection models in recent years. YOLOv5 [13] is renowned for its fast processing speeds, making it suitable for real-time detection tasks. However, its accuracy is not high in detecting very small or highly occluded objects. YOLO7 [14] and IYOLO7 [15] provide improvements in detection accuracy and speed, particularly in handling complex object detection scenarios with better handling of occlusions and small objects. However, the increased complexity leads to longer training times, and YOLO7 and IYOLO7 require more computational power for optimal performance.

The development of image detection models based on CNN has made significant progress, especially in the context of drone images. However, CNN models also face some challenges, such as scale sensitivity. CNN can struggle with scale variations within an image because they process information at predefined scale levels. Additionally, the layered structure of CNN requires the output of one layer to complete before the next can begin, which can lead to inefficiencies in processing time. Therefore, CNN cannot meet the demand for real-time detection of multi-scale images. Models based on transformer or combining CNN with transformer have become research directions in recent years.

### 2.2 Transformer based object detection technology

In recent years, Transformer have been widely used in the field of small object detection. Carion et al. introduced DETR [5], an end-to-end system simplifying target detection into a direct set prediction problem. It utilizes the deep relational modeling capabilities of Transformers for object queries and pairs sample matching and box positioning with bipartite graph matching through the Hungarian algorithm. However, DETR still has shortcomings in convergence speed and detection performance of small objects. To address this, Zhu et al. adopted a deformable self-attention mechanism [16], which enhances the recognition of small objects by strategically sampling key areas of the image and fusing multi-scale features. Dai et al. further developed dynamic attention techniques [17], dynamically adjusting according to the importance of scale, spatial location, and feature dimensions to optimize performance and accelerate model convergence. However, their methods may cause feature confusion during self-attention computations by merging features of different scales into a single input for the encoder.

In the single-stage models of DETR, object queries are randomly initialized. DINO [18] enhances position queries by selecting top-K features from the Encoder output, enriching the prior information of object queries. RT-DETR [19] adopts an IoU-aware query selection method, picking high-scoring feature samples to optimize the Decoder’s input. However, selecting a fixed number of feature samples may limit the Decoder’s feature expression and cause sparse supervision in the Encoder’s optimization process.

Furthermore, some studies focus on the instability issues of the Hungarian algorithm, reflected in different prediction boxes being assigned to the same true target across training epochs. Yuan [20] proposed Parallel Attention-MLP for insulator defect detection (PAM-DETR). Within the parallel branches, one branch employs a modified self-attention mechanism to capture long-range dependencies, while the other branch extracts local token relationships using MLP networks.

The above studies are based on DETR and have been modified to address specific weaknesses such as slow convergence time and difficulty in detecting small objects. This is precisely the research direction of DETR’s industrial applications. This study proposes an improved multi-scale DETR design for insulator defect detection, which achieves cross scale local attention in different paths of the feature pyramid.

### 2.3 Detection of small objects

Current technologies for detecting small objects primarily modify and optimize standard object detection frameworks to accommodate small-sized targets. These techniques can generally be classified into four mainstream directions based on their improvement strategies:

1. Data augmentation methods are crucial for training data-driven deep learning models, especially in the field of small object detection where standard sampling techniques often fail to provide sufficient training samples [21]. One approach is to replicate and randomly transform small objects within the same image, enlarging the sample size of small objects and enhancing the model’s detection capability. Another strategy involves new label assignments to include more small objects in training.
2. Multi-scale fusion methods allow for recognition by high-level features in deep networks, which are typically small in scale and do not encompass low-level features [22]. Considering that small object features are often present in low-level features, PANet [23] leverages the concept of inter-layer feature interaction from FPN, enhancing feature hierarchy through bi-directional paths, thus improving the precision of deep feature localization. Yang et al. [24] adopted Scale-Dependent Pooling (SDP) to choose appropriate feature layers for the pooling steps of small objects. MS-CNN [25] generates object proposals for specific scale ranges, providing optimal receptive fields for small objects.
3. Attention mechanism methods [26] mimic the human ability to rapidly scan scenes while ignoring irrelevant parts, with our perceptual system utilizing visual attention mechanisms crucially during recognition. Attention models assign different weights to different parts of the feature map, highlighting important areas while suppressing secondary information.
4. Contextual information methods utilize spatial correlations, providing additional clues and evidence. Contextual information plays a crucial role in human visual systems and scene understanding tasks. Some methods enhance the detection of small objects by leveraging contextual clues. Chen et al. [27] used context representations encompassing the proposal region for recognition. Hu et al. [28] explored encoding areas beyond the object range and modeled local context information in a scale-invariant manner to detect small objects. Based on this, we combine contextual information with attention to better distinguish the relationships between foreground and background.

## 3. Method

### 3.1 Overview

**Fig 1:**
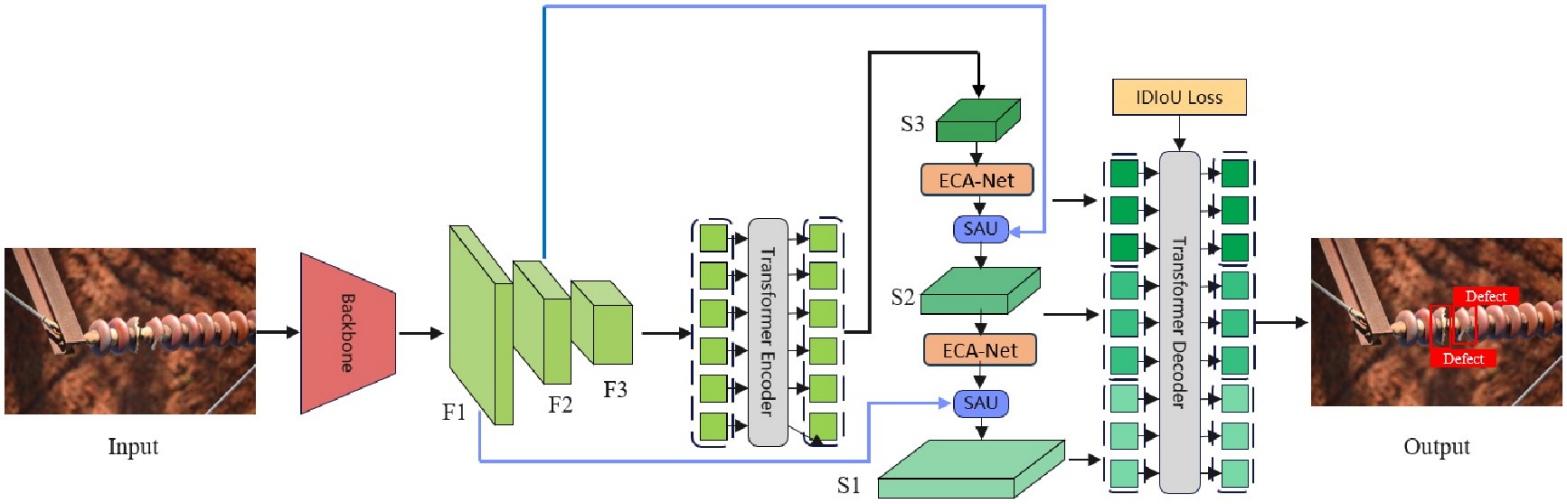
IF-DETR model.

The aim of this research is to utilize the DETR architecture for the identification of defects on insulators, with a special emphasis on enhancing the detection precision of minute flaws. As demonstrated in Fig 1, the IF-DETR(Insulators Flaws DETR) model is an end-to-end crater identification model based on Transformer technology.

There are two innovations in this model, one is the fusion of low-level features from the backbone network with high-level features from the Transformer through the fusion module of ECA-Net and SAU to further capture contextual information. The other is to solve the instability problem caused by small defects in the matching process through IDIoU loss function and to speed up the training.

This model employs a multi-scale feature fusion approach, integrating features from before and after the encoder at various scales. Efficient Channel Attention (ECA-Net) [29] is an attention mechanism focused on the channel dimension. It enhances feature representation through local cross-channel interactions without significantly increasing computational overhead. SAU (Spatial Attention Upsampling) [30] focuses on feature upsampling and cross-scale fusion through a spatial attention mechanism. Combining these two techniques optimizes feature representation at different levels, improving the model’s overall performance. In the Head section, multi-scale features processed by cross-scale fusion are fed into the transformer decoder, thus increasing the quantity of queries involved in training. Ultimately, to simplify the optimization of small objects, we have designed a specialized stable IoU(Intersection over Union) loss function [31] for small objects.

Multi-scale Architecture In convolutional neural networks, it has been proven by Cai [32] that multi-scale feature fusion can effectively improve detection accuracy. However, the increased length of input sequences still leads to significant computational demands. To overcome this obstacle, we rethought the encoder structure and designed a series of variants with different encoders, as shown in Fig 1. Feature fusion at two scales not only achieves good detection results but also avoids introducing excessive computation. Applying self-attention to high-level features with richer semantic concepts can capture the relationships between conceptual entities within images, aiding subsequent modules in the detection and identification of objects in images. Moreover, the fusion of features across multiple levels can effectively extract contextual information around defects to prevent confusion between the foreground and background.

### 3.2 Efficient channel attention network

**Fig 2:**
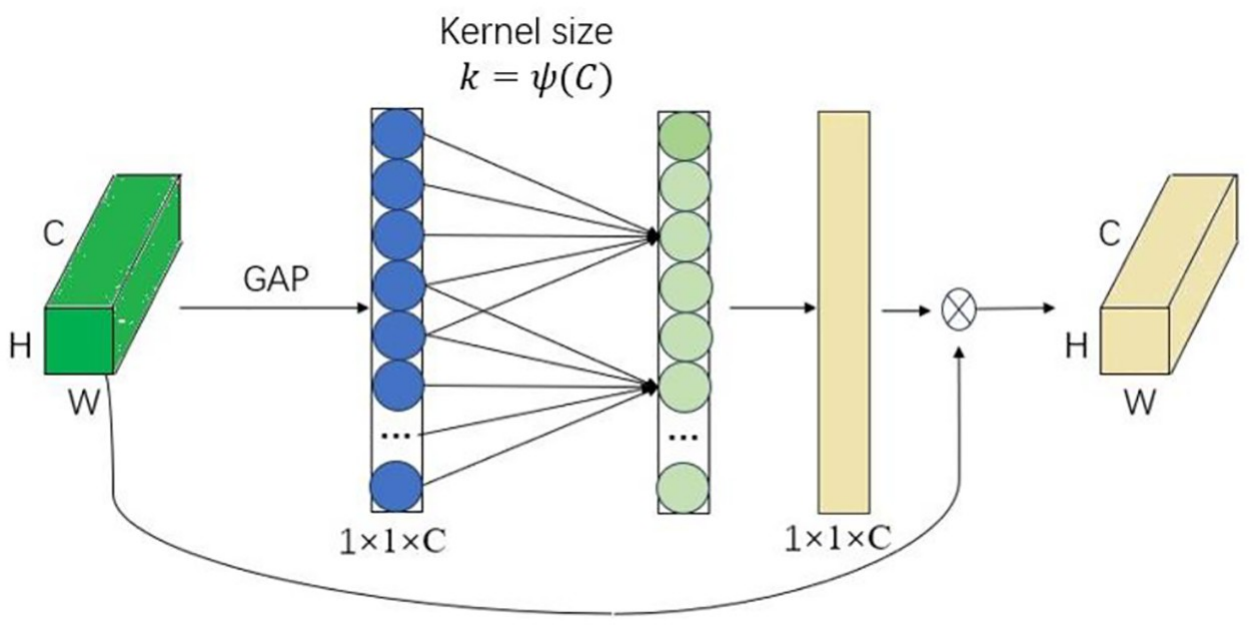
Efficient channel attention network module.

CNN tends to treat all feature channels in an image equally, ignoring the correlations between different channels. This can lead to interference from redundant information when extracting features, reducing performance. To address this issue, the attention mechanism was introduced, allowing the network to dynamically adjust its focus on different feature channels. ECA-Net is a model based on the attention mechanism that enhances the network’s feature modeling capability by introducing channel attention, as shown in Fig 2.

Given the aggregated features obtained by global average pooling (GAP), ECA-Net generates channel weights by performing a fast 1D convolution of size k, where k is adaptively determined via a mapping of channel dimension C.

The core idea of ECA-Net is to introduce channel attention after the convolutional layer to dynamically adjust the response of different channels. First, ECA-Net uses GAP to reduce the dimensionality of the feature map. Then, the output from the global average pooling is reduced to a smaller vector via a fully connected layer. This vector captures the global information of each channel. Finally, the output of the channel attention module is connected to the input of the convolutional layer using a residual connection to ensure lossless information transfer.

### 3.3 Self-attention upsampling

**Fig 3:**
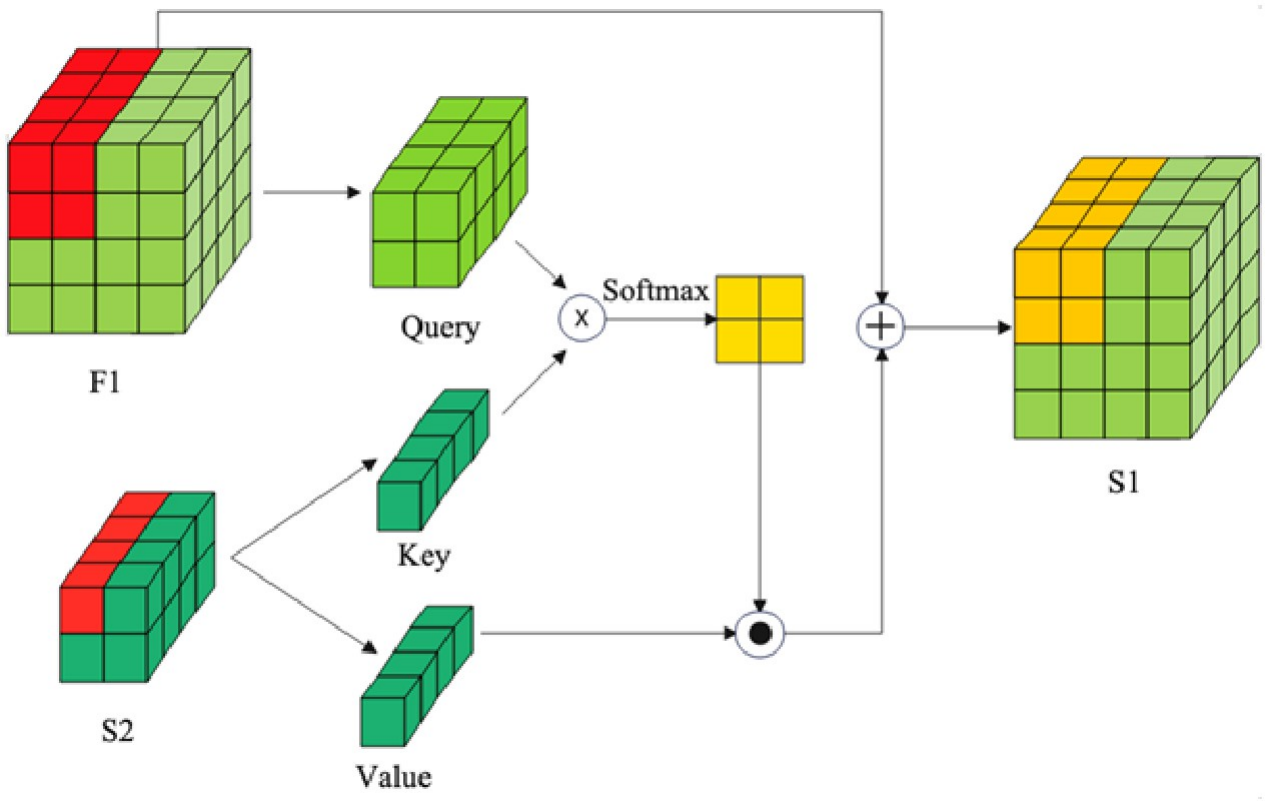
Self-attention upsampling layer.

During feature fusion, traditional upsampling methods primarily include interpolation algorithms and deconvolution. Interpolation has limitations in capturing rich semantic information, and deconvolution is limited due to the use of a uniform kernel. To address this, CARAFE(Content-Aware ReAssembly of FEatures) [33] introduced a novel convolutional upsampling method, which reassembles features in a content-aware manner. However, this approach only integrates high-level features into low-level features without addressing cross-scale feature interactions. We propose a self-attention upsampling layer, as shown in Fig 3. This layer utilizes cross-scale local cross-attention, where each element in the low-resolution feature map calculates cross-attention with the corresponding high-resolution feature region. This ensures semantic consistency and enhances feature expressiveness by fusing high and low-level features. Moreover, it effectively preserves the spatial information of small defects and their context in low-level features, preventing foreground-background confusion.

High-level features S_2_ ∈ R^c×h/2×w/2^ are enhanced by the features F_1_ ∈ R^c×h/2×w/2^ from the transformer encoder, and the high-resolution low-level features F_1_ ∈ R^c×h×w^ are extracted from the backbone network. A “2 × 2” sliding window with a stride of 2 is used. As the sliding window moves across F_1_ once, a *Query*_*i*_ ∈ R^c×w×w^ is obtained through the self-attention mechanism. Meanwhile, the i-th feature pixel in S is considered as *Key*_*i*_ ∈ R^c×l×l^ and Value_*i*_ ∈ R^c×l×l^. After linearly projecting *Query*_*i*_ and *Key*_*i*_, *Q*_*i*_ and *K*_*i*_ are obtained. The Key and Value with index i correspond to the Query with index i. The i-th attention weight unit A_i_ is defined as the dot product of *Q*_*i*_ and *K*_*i*_. The final weights are obtained using a Softmax operation and are then used to perform a dot product with features S_2_ and fuse with low-level features F to obtain the final result S.

The calculation of the weights is shown in Eq 1 [34]:

$$A_{i}=Q_{i}K_{i}^{T},\;W=softmax\left(\frac{Ai}{\sqrt{d}}\right)$$ (1)

where d is the normalization factor.

The computation is defined by Eq 2:

$$V={\left[V_{0},\;V_{1},\;\ldots ,\;V_{\frac{hw}{4}}\right]}^{T}$$ (2)

In the matrix V, each row is populated with the projection of the i-th high-level feature pixel, denoted as Vi.

The resulting feature matrix S is obtained as shown in Eq 3:

S=V⊙W+F (3)

This study combines ECA-Net and SAU to form a fusion module. ECA-Net enhances the expressive power of features by using local cross channel interactions, while SAU focuses on feature upsampling and cross scale fusion through spatial attention mechanisms. The combination of the two can optimize feature representation at different levels, thereby improving the overall performance of the model.

### 3.4 Insulator defect IDIoU loss function

The loss function for two-stage DETR variants typically comprises three components, classification loss, box L1 loss, and IoU-based loss. The loss function of IF-DETR is defined as follows:

$$L\left(y,y^{gt}\right)=\lambda_{cls}L_{cls}\left(c,\;c^{gt}\right)+\lambda_{l1}∥b-b^{gt}{∥}_{1}+\lambda_{\text{IDIoU}}L_{\text{IDIoU}}{\left(b,\;b^{gt}\right)}^{\prime }$$ (4)

Hyperparameters are utilized to modulate the scaling of the three constituent parts of the loss. Additionally, y = [1] and *y*^*gt*^ = {*c*^*gt*^, *b*^*gt*^} respectively denote the prediction and ground truth, where c and b represent the class and bounding box. *L*_*cls*_() and *L*_*IDIoU*_() are the classification loss and IDIoU loss that we have designed to optimize the difficulty associated with defect detection. The classification loss employs Focal loss is defined as follows:

$$L_{cls}\left(c,\;c^{gt}\right)=\left\{\begin{array}{rr} -{\left(1-c\right)}^{\gamma }logc, & if\;c^{gt}=1\\ -c^{\gamma }\text{log}\left(1-c\right), & if\;c^{gt}=0 \end{array}\right.$$ (5)

We have observed that optimizing small defect detection during the training process presents challenges, primarily due to the defect features being easily conflated with background features. Furthermore, enhancing stability in one-to-one matching is also challenging. The original DETR uses IoU loss to measure the overlap between predictions and ground truth, but IoU loss does not accurately reflect the distance between prediction boxes and actual boxes. Consequently, many DETR variants have opted for GIoU(Generalized Intersection over Union) loss [6] in place of IoU loss. However, when two boxes have a containment relationship, GIoU loss degrades into IoU loss, which does not convey the boxes’ relative positions, disadvantaging datasets with densely packed small defects. For example, during the initial stages of training, a single predicted box may encompass multiple small defects. In such cases, GIoU loss contributes little to one-to-one matching, whereas classification loss and box L1 loss become more significant. Additionally, we found that one-to-one matching using only classification scores and L1 scores yields disparate outcomes, leading to instability in the matching process as the model does not optimize towards a unified objective.

To address these issues, we propose the IDIoU loss for small defects, based on EIoU(Efficient-IoU) Loss [35]. EIoU Loss consists of three components: IoU loss, center distance loss, and aspect ratio loss. We found that even if the predicted box is within the actual box, the EIoU Loss can still effectively discriminate the predicted box’s relative position and incorporates the supervision of the box L1 loss into the IoU loss. Our specially designed IDIoU Loss for small defects uses classification scores, and optimizing all components in a unified direction. We introduce the Defect IoU Loss to accommodate this enhancement, as illustrated by Eq 6:

$$L_{\text{IDIoU}}=1+rIoU-IoU+\frac{\rho^{2}\left(o,o^{gt}\right)}{{\left(w^{c}\right)}^{2}+{\left(h^{c}\right)}^{2}}+\frac{\rho^{2}\left(w,w^{gt}\right)}{{\left(w^{c}\right)}^{2}}+\frac{\rho^{2}\left(h,h^{gt}\right)}{{\left(h^{c}\right)}^{2}}$$ (6)

where o and *o*^*gt*^ denote the central of prediction box and ground truth box, *w*^*c*^ and *h*^*c*^ are the width and height of the smallest enclosing box of the two boxes. *ρ*^2^ () represents the Euclidean distance between two points. r represents the classification scores.

## 4. Experiment

### 4.1 Dataset

In the realm of insulator fault detection, data are relatively scarce, and research typically relies on proprietary datasets. Presently, there are two mainstream publicly available datasets: The Chinese Power Line Insulator Dataset and the augmented version of the same dataset. We conducted comparative experiments on these two datasets against state-of-the-art methods and performed ablation studies on the backbone network structure, loss function selection, and upsampling mechanism choice. As the primary objective of this paper is to enhance detection accuracy, our experiments primarily utilize recall and precision as evaluation metrics.

- **IFDD Dataset**

**Fig 4:**
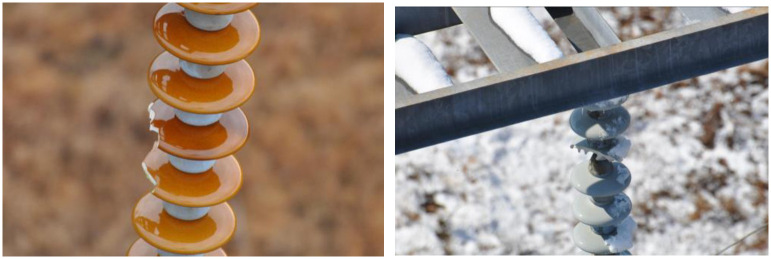
Samples of the IFDD dataset.

The IFDD dataset includes 1600 image samples, which are divided in a 7:2:1 ratio. It encompasses a sufficient number of defect samples, covering most of the defect scenarios found in insulators in daily life, with a wide distribution of data. The samples of the IFDD Dataset are shown in Fig 4. The dataset contains data of various sizes, predominantly small and medium-sized targets, which aligns more closely with real-world scenarios.

- **CPLID Dataset**

**Fig 5:**
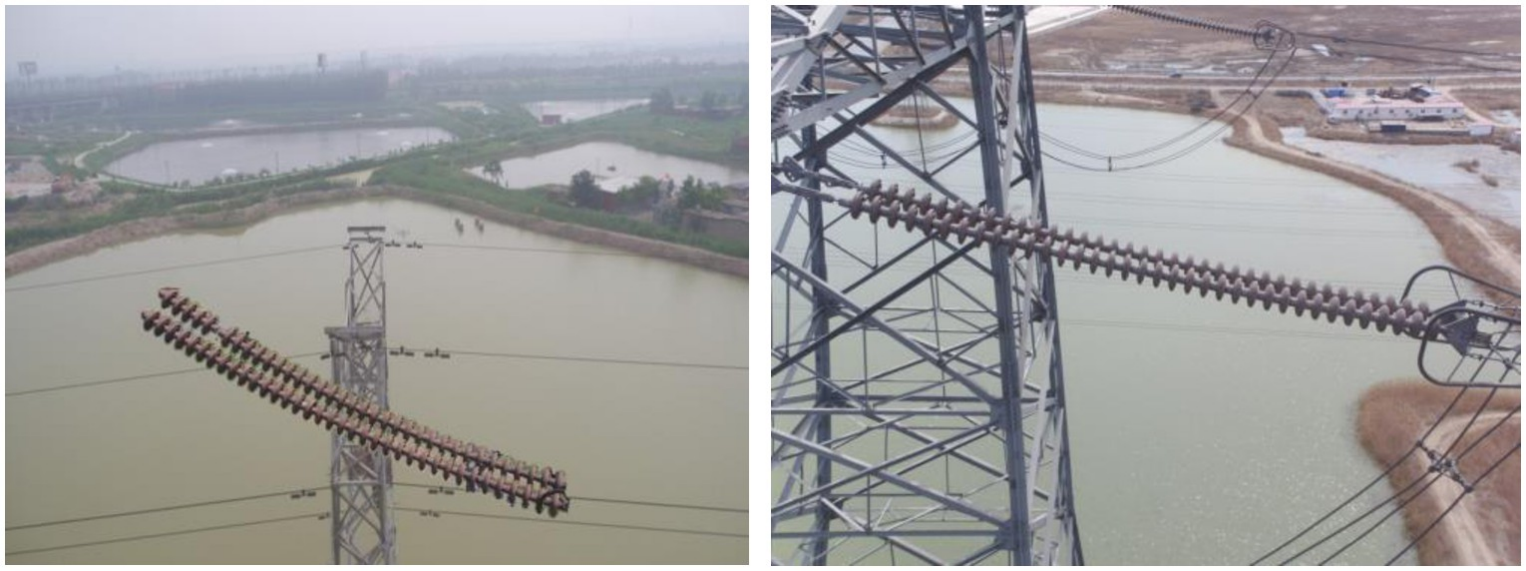
Samples of the CPLID dataset. (Reprinted from [36] under a CC BY license, with permission from Z, Wang, original copyright 2018).

The Chinese Power Line Insulator Dataset (CPLID) comprises images of normal and defective insulators captured by Unmanned Aerial Vehicles (UAVs) for the State Grid Corporation of China [36]. Specifically, CPLID contains 600 images of normal insulators and 248 images of defective insulators. The samples of the CPLID Dataset are shown in Fig 5. Given the limited number of defective insulator images, some were generated by segmenting defective insulators and superimposing them onto different backgrounds.

### 4.2 Training settings

In our experiments, we utilized a Resnet model [37] pre-trained on ImageNet as the backbone network for extracting multi-scale features. We adopted a linear warm-up strategy for learning rate optimization, settling on a final learning rate of 0.0002. In addition, the loss function was optimized over 200 epochs using the AdamW optimizer with a weight decay of 0.0001. The anchors within the auxiliary head were aligned with those used in YOLOv3 [38]. All experiments were implemented in PyTorch and carried out on a Tesla A100 GPU.

### 4.3 Evaluation metrics

To assess the detection quality of our model, we used precision and recall as performance metrics on the dataset, similar to most detection methods. We categorized predicted boxes with an IoU greater than 0.5 compared to the ground truth as true positives and the rest as false positives. The specific formulas are as follows:

$$Precision=\;\frac{\left\|TP\right\|}{\left\|TP\right\|+\left\|FP\right\|}$$ (7)

$$Recall=\;\frac{\left\|TP\right\|}{\left\|TP\right\|+\left\|FN\right\|}$$ (8)

Prediction boxes that do not contain defects are classified as false negatives (FN). Prediction boxes that contain defects and have an IoU greater than 0.5 with the ground truth are classified as true positives (TP), while those with an IoU less than or equal to 0.5 are classified as false positives (FP). ‖TP‖, ‖FP‖, and ‖FN‖ represent the counts of true positives, false positives, and false negatives respectively. Higher accuracy and recall rates indicate better detection performance.

### 4.4 Compared with other methods

On the IFDD dataset, we compared the IF-DETR with other advanced DETR variants. Table 1 displays the results of these comparisons. It is evident from the table that our model achieved the highest precision and recall with fewer parameters and made progress in detecting small defects (APs).

**Table 1:** Comparion of different methods in IFDD dataset.

Model	Backbone	#Epcohs	#Param(M)	FLOPS	Precision(%)	Recall(%)	APs(%)
Faster-RCNN [39]	R50	24	-	-	80.5	65.2	29.5
ATSS [40]	R50	24	-	-	80.6	70.3	32.5
YOLOv5 [13]	-	275	-	-	81.3	67.7	28.5
YOLOv7 [14]	-	275	-	-	89.5	86.7	30.2
IYOLOv7 [15]	-	275	-	-	91.1	88.4	34.3
Deformable-DETR [41]	R50	50	40.2	170.1	81.2	81.5	26.2
Conditional-DETR [42]	R50	50	43.3	63.7	82.1	68.3	25.5
Group-DETR [43]	R50	50	44.1	63.2	78.3	78.2	31.6
Focus-DETR [44]	R50	36	49.5	113.6	88.1	83.1	33.4
DINO [18]	R50	12	47.6	178.4	89.8	83.4	33.7
RT-DETR [28]	R50	72	41.4	93.4	90.6	87.5	34.1
**IF-DETR(Ours)**	**R50**	**72**	**39.3**	**90.1**	**92.3**	**89.6**	**35.2**

Note: The missing part of the data is based on CNN methods, which are not comparable to those based on DETR methods.

Compared to high-performance models like RT-DETR [28], Focus-DETR [44], and DINO [18], our method averaged a 3% improvement in detection accuracy. These models struggle to identify defects with weak features or in complex backgrounds because they unify multi-scale features into a single input for the encoder, leading to increased computational cost and potential feature confusion. In contrast, our model integrates a feature fusion step between the encoder and decoder, effectively ensuring that features of small craters in high-resolution feature maps are not overlooked during the learning process. Additionally, our training strategy ensures that the encoder learns higher-quality features.

Compared to the baseline model RT-DETR, IF-DETR showed more than a 2% increase in precision. This is due to the fact that RT-DETR’s interpolation upsampling and convolutional pooling fail to ensure semantic coherence of features. Moreover, RT-DETR’s sparse sampling method for object query selection limits the decoder’s decoding capabilities. Our proposed SAU operator ensures the consistency of upsampled features, suitable for networks based on self-attention computation.

**Fig 6:**
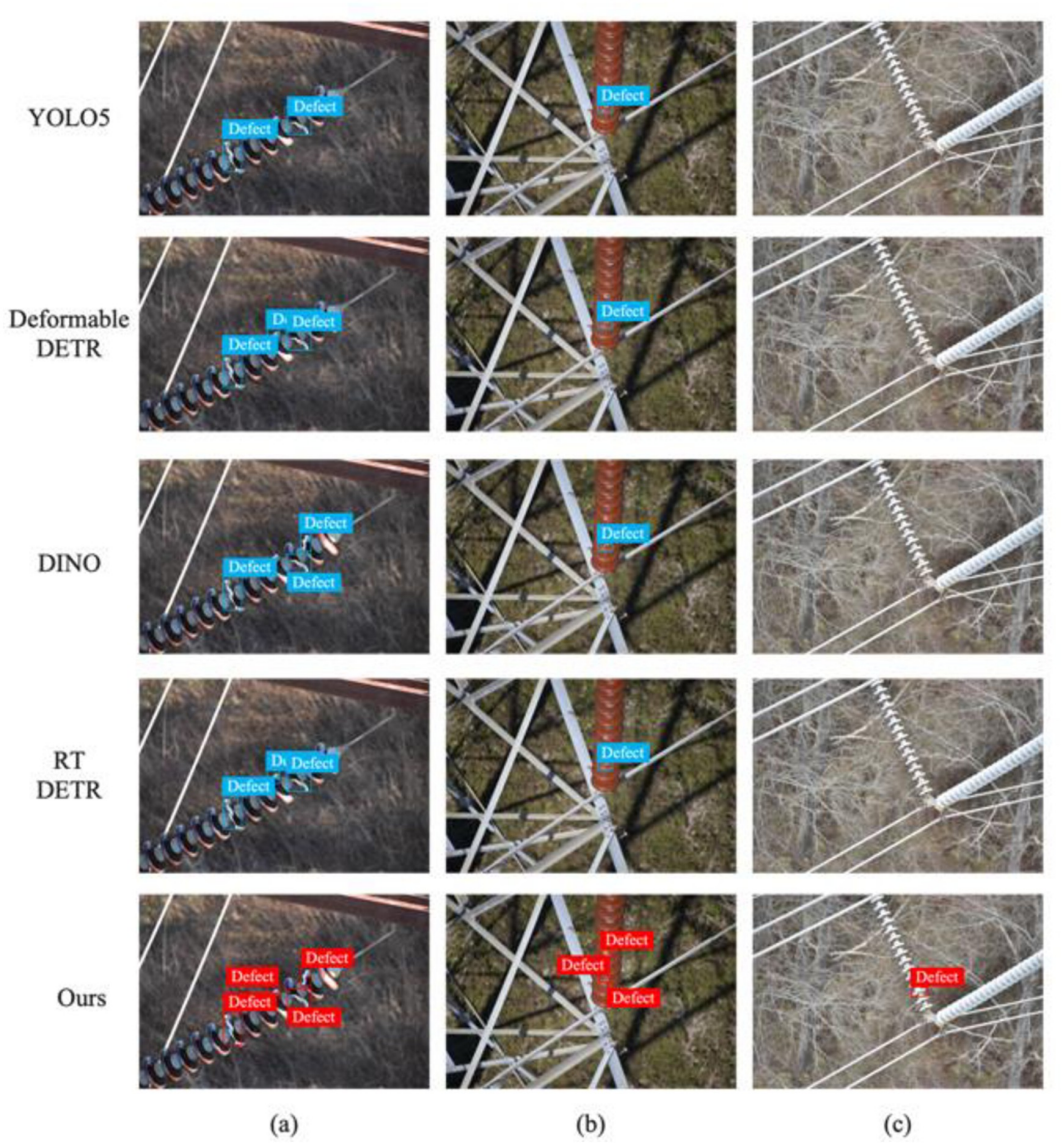
The detection results of different methods. (Reprinted from [36] under a CC BY license, with permission from Z, Wang, original copyright 2018).

Fig 6 showcases the detection results of four high-performance DETR variants and our proposed method. The results indicate that Deformable-DETR and RT-DETR underperform in identifying small defects, with instances of missed detections as shown in the second column of Fig 6. Our algorithm’s precise design effectively identifies small craters with less evident edge features, thus demonstrating exceptional detection performance on such objects.

In the third column of Fig 6, our model displays an advantage in handling images with complex backgrounds. The complex texture features in these images pose a challenge to detection as they resemble defects. Although complex backgrounds challenge all detectors, our method is least affected and accurately detects defects where other methods fail, benefitting from our model’s proposed self-attention upsampling module which effectively differentiates between foreground and background, resulting in superior feature learning capabilities and enhanced robustness of the model.

**Fig 7:**
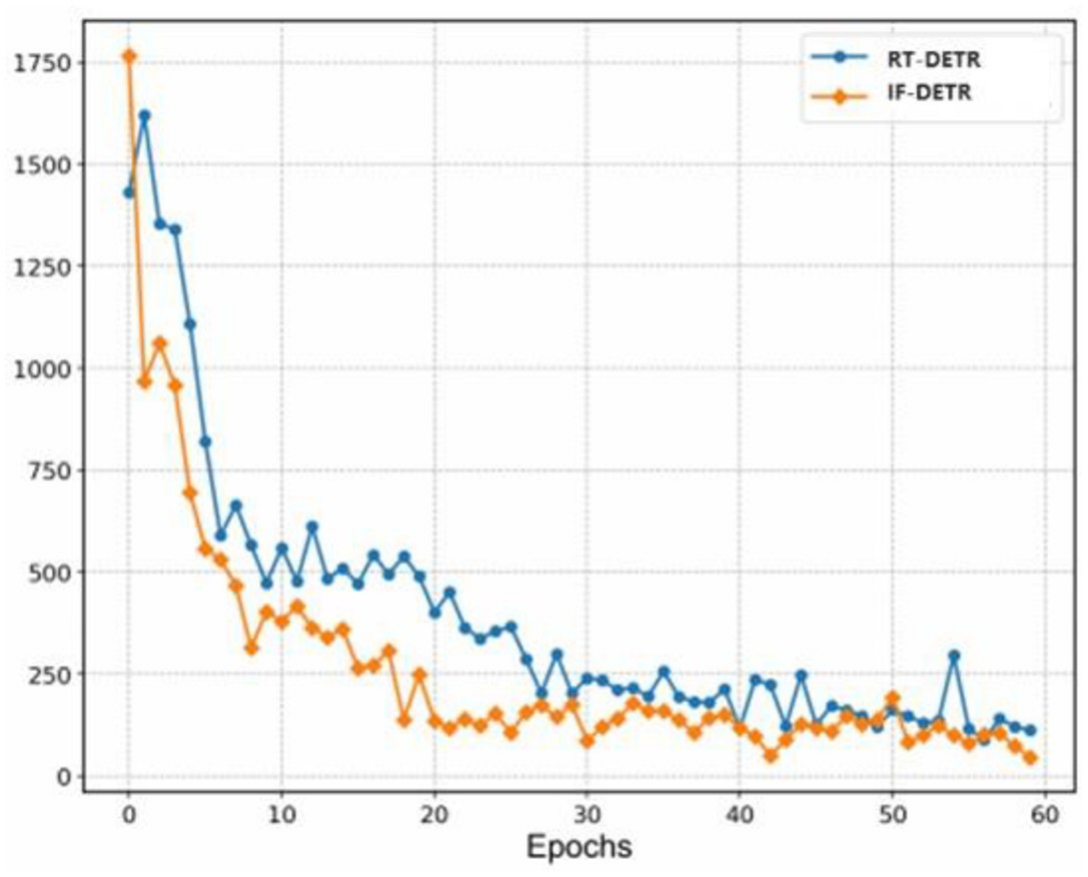
Comparison of RT-DETR and IF-DETR instability scores on small defect.

In Fig 7, we make a separate comparison for small defects. Compared with the baseline model, our model’s instability score drops faster in the early stages of training, and it is more stable in the later stages of training. It could present that our method effectively mitigates the instability of small defect matching and accelerates the optimization process.

Overall, our algorithm has clear advantages in feature learning and distinguishing between foreground and background features. It improves the accuracy of defect detection, especially for small defects, through the use of more effective multi-scale feature fusion techniques and a more stable matching process.

## 5 Discussion

### 5.1 Ablation study

We conducted a series of ablation experiments to assess the specific contributions of each component to the model’s performance. By incrementally incorporating the three innovative points we proposed into the robust baseline model, the detection results improved. These experimental results are summarized in Table 2. Initially, we utilized a multi-scale backbone network structure within the classic DETR, which improved the precision of defect detection by 0.9%, underscoring the benefits of multi-scale feature fusion under the DETR framework. Subsequently, by integrating the fusion module of ECA-Net and SAU into the multi-scale bottom-up path, the model’s detection precision increased by 0.4%, reflecting the efficacy of the fusion module in emphasizing small defect features. The model’s performance increased by 0.4%, demonstrating the appropriate upsampling enhancement of the model’s ability to differentiate foreground from background. Finally, by replacing the original loss function with the IDIoU loss, the model’s detection precision increased again by 0.4%, indicating the beneficial impact of IDIoU loss on network training. In summary, our experiments demonstrate that each component of the proposed algorithm effectively enhances the model’s performance, and they work in coordination without any conflict.

**Table 2:** Influence of each component.

Model	Multi-scale	ECA+SAU	IDIoU	Precision (%)	Recall (%)
Baseline	-	-	-	90.6	87.5
Baseline + Parts	√	-	-	91.5 ↑0.9	88.3 ↑0.8
	√	√	-	91.9 ↑1.3	88.9 ↑1.4
	√	√	√	92.3 ↑1.7	89.6 ↑2.1

### 5.2 Generalization study

To further validate the robustness and broad applicability of the proposed model in handling insulator defect detection, we conducted experiments on a subset of the CPLID dataset. According to the results in Table 3, our model continues to demonstrate exceptional detection capabilities. From the experimental results, the defects in the CPLIID data are relatively simple, and existing algorithms have very high detection accuracy. Compared with the best algorithm, Hybrid-YOLO [45], our method is comparable in precision, but we have improved by 2.6% in recall rate. This indicates that our method can identify as many defects as possible, thanks to a series of innovative points we proposed, which are more conducive to actual industrial applications. In addition, compared with DETR-based methods, our method also has significant improvements, which shows that our method has strong robustness and can achieve better experimental results across multiple data distributions.

**Table 3:** Comparion of different methods in CPLID dataset.

Model	Precision(%)	Recall(%)
**YOLO-5** [13]	95.2	96.3
**Hybrid-YOLO** [45]	99.4	93.1
**Deformable-DETR** [41]	90.2	84.4
**Focus-DETR** [44]	91.5	85.2
**DINO** [18]	97.1	92.1
**RT-DETR** [28]	98.3	93.6
**IF-DETR(Ours)**	**99.2**	**95.7**

### 5.3 Practical and social implications

Addressing the challenges in detecting insulator defects have significant practical and social implications. First, we proposed a context-based attention module to enhance detection precision. By focusing on the context, the model can distinguish insulator defects from similar background features more accurately, leading to reduced false positives and negatives. This precision is crucial for maintaining power line integrity and reducing the risk of power outages. Improved detection accuracy enables utility companies to more effectively prioritize maintenance and repair efforts, focusing resources on areas with actual defects, thus optimizing operational costs. Second, the IDIoU loss function’s ability to optimize the detection of small defects is particularly valuable for early-stage defect identification. Catching minor defects before they evolve into larger issues can prevent significant damage. Faster training speeds enabled by the IDIoU loss function allow for more rapid development and deployment of defect detection models, facilitating timely updates and improvements to the detection system.

In terms of social significance, accurate identification of defects contributes to the timely repair of potential hazards, significantly reducing the risk of accidents caused by equipment failure, such as fires or power outages. Enhancing the reliability of power infrastructure through better defect detection ensures a more consistent power supply, which is essential for both residential and commercial consumers. Early detection and repair of minor defects can significantly reduce the costs associated with large-scale repairs and power outages, contributing to economic stability by avoiding unexpected expenses for utility companies and consumers.

In summary, the proposed context-based attention module and IDIoU loss function not only address specific technical challenges in the field of insulator defect detection but also contribute to broader practical and social benefits. These include enhancing the safety, reliability, and sustainability of power infrastructure, which are essential components of modern society’s functioning and well-being.

## 6. Conclusion

In this study, we developed a variant of the DETR model, named IF-DETR, specifically for the detection of insulator defects. The core objective of this model is to enhance the precision of small defect detection. Initially, we integrated a multi-scale backbone network with the classical DETR framework, which bolstered the features of small defects to some extent. Subsequently, we introduced a fusion module of ECA-Net and SAU, based on cross-scale local cross-attention computation. These operators are applied in the bottom-up path of the multi-scale backbone network for the fusion of features at various scales, effectively addressing the issue of distinguishing small defects from the background. Moreover, to address the instability of one-to-one matching for small craters, we designed a new loss function named IDIoU loss. This loss function calculates matching cost and box regression loss, significantly improving the stability of small defect matching.

Compared to existing CNN-based defect detection algorithms, our method has significantly increased detection accuracy. It has demonstrated excellent feature representation capabilities and robustness, especially for small defects in complex backgrounds.

While certain results have been achieved, there is still room for improvement in our work. The current dataset limitations hinder the effective detection of internal crack defects in insulating materials. In the future, we plan to expand the existing dataset to facilitate the detection of such defects.
